# Pan‐Arctic soil moisture control on tundra carbon sequestration and plant productivity

**DOI:** 10.1111/gcb.16487

**Published:** 2022-11-10

**Authors:** Donatella Zona, Peter M. Lafleur, Koen Hufkens, Beniamino Gioli, Barbara Bailey, George Burba, Eugénie S. Euskirchen, Jennifer D. Watts, Kyle A. Arndt, Mary Farina, John S. Kimball, Martin Heimann, Mathias Göckede, Martijn Pallandt, Torben R. Christensen, Mikhail Mastepanov, Efrén López‐Blanco, Albertus J. Dolman, Roisin Commane, Charles E. Miller, Josh Hashemi, Lars Kutzbach, David Holl, Julia Boike, Christian Wille, Torsten Sachs, Aram Kalhori, Elyn R. Humphreys, Oliver Sonnentag, Gesa Meyer, Gabriel H. Gosselin, Philip Marsh, Walter C. Oechel

**Affiliations:** ^1^ Department Biology San Diego State University San Diego California USA; ^2^ School of Biosciences University of Sheffield Sheffield UK; ^3^ School of the Environment Trent University Peterborough Ontario Canada; ^4^ BlueGreen Labs Melsele Belgium; ^5^ National Research Council (CNR) Institute of BioEconomy (IBE) Florence Italy; ^6^ Department of Mathematics and Statistics, San Diego State University San Diego California USA; ^7^ LI‐COR Biosciences Lincoln Nebraska USA; ^8^ The Robert B. Daugherty Water for Food Global Institute and School of Natural Resources University of Nebraska Lincoln Nebraska USA; ^9^ University of Alaska Fairbanks Fairbanks Alaska USA; ^10^ Woodwell Climate Research Center Falmouth Massachusetts USA; ^11^ W.A. Franke College of Forestry & Conservation The University of Montana Missoula Montana USA; ^12^ Max Planck Institute for Biogeochemistry Jena Germany; ^13^ Faculty of Science, Institute for Atmospheric and Earth System Research (INAR) / Physics, University of Helsinki Helsinki Finland; ^14^ Department of Ecoscience, Arctic Research Centre Aarhus University Roskilde Denmark; ^15^ Oulanka Research Station Oulu University Kuusamo Finland; ^16^ Department of Environment and Minerals, Greenland Institute of Natural Resources Nuuk Greenland; ^17^ Royal NIOZ the Netherlands Institute for Sea Research Texel Netherlands; ^18^ Department of Earth and Environmental Sciences, Lamont‐Doherty Earth Observatory Columbia University Palisades New York USA; ^19^ Jet Propulsion Laboratory California Institute of Technology Pasadena California USA; ^20^ Environmental Meteorology, Institute of Earth and Environmental Sciences University of Freiburg Freiburg Germany; ^21^ Institute of Soil Science, Center for Earth System Research and Sustainability (CEN) Universität Hamburg Hamburg Germany; ^22^ Geography Department Humboldt‐Universität zu Berlin Berlin Germany; ^23^ Alfred Wegener Institute Helmholtz Centre for Polar and Marine Research Potsdam Germany; ^24^ GFZ German Research Centre for Geosciences Potsdam Germany; ^25^ Department of Geography & Environmental Studies Carleton University Ottawa Ontario Canada; ^26^ Département de Géographie Université de Montréal Montréal Quebec Canada; ^27^ Department of Geography and Environmental Studies, Wilfrid Laurier University Waterloo Ontario Canada

**Keywords:** carbon loss, climate change, drying, permafrost, tundra

## Abstract

Long‐term atmospheric CO_2_ concentration records have suggested a reduction in the positive effect of warming on high‐latitude carbon uptake since the 1990s. A variety of mechanisms have been proposed to explain the reduced net carbon sink of northern ecosystems with increased air temperature, including water stress on vegetation and increased respiration over recent decades. However, the lack of consistent long‐term carbon flux and in situ soil moisture data has severely limited our ability to identify the mechanisms responsible for the recent reduced carbon sink strength. In this study, we used a record of nearly 100 site‐years of eddy covariance data from 11 continuous permafrost tundra sites distributed across the circumpolar Arctic to test the temperature (expressed as growing degree days, GDD) responses of gross primary production (GPP), net ecosystem exchange (NEE), and ecosystem respiration (ER) at different periods of the summer (early, peak, and late summer) including dominant tundra vegetation classes (graminoids and mosses, and shrubs). We further tested GPP, NEE, and ER relationships with soil moisture and vapor pressure deficit to identify potential moisture limitations on plant productivity and net carbon exchange. Our results show a decrease in GPP with rising GDD during the peak summer (July) for both vegetation classes, and a significant relationship between the peak summer GPP and soil moisture after statistically controlling for GDD in a partial correlation analysis. These results suggest that tundra ecosystems might not benefit from increased temperature as much as suggested by several terrestrial biosphere models, if decreased soil moisture limits the peak summer plant productivity, reducing the ability of these ecosystems to sequester carbon during the summer.

## Graphical abstract

Long‐term atmospheric CO_2_ concentration records suggested a reduction in the positive effect of warming on high‐latitude carbon uptake, potentially linked to water stress on vegetation or increased respiration. The lack of data has limited our ability to identify the mechanisms responsible for the reduction in the positive effect of warming. This study, using a record of 100 site‐years of data from 11 sites distributed across the Arctic, showed a decrease in plant productivity with increased temperature during the peak summer‐related soil moisture limitation. Tundra ecosystems might not benefit from warming if moisture stress limit carbon sequestration during the summer.

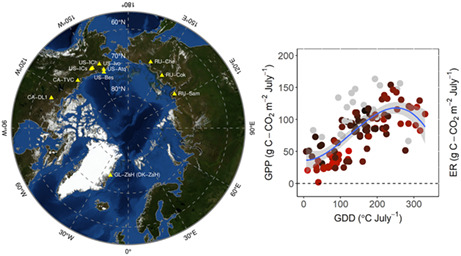

## 1 INTRODUCTION

Satellite observations from the late 20th century and early 21st century suggested that plant productivity increased widely in northern high latitudes in response to warming (Berner et al., 2020; Guay et al., 2014; Myneni et al., 1997; Nemani et al., 2003). Yet, recent large‐scale analyses based on atmospheric CO_2_ measurements from the Arctic suggest that summer CO_2_ uptake has been waning in response to rising air temperatures in high‐latitude ecosystems over the last few decades (Piao et al., 2014; Wang et al., 2018). Water stress on vegetation could be one of the possible mechanisms explaining the decrease in the positive response of plant productivity to warmer temperatures (Angert et al., 2005; Piao et al., 2014; Wang et al., 2018), as water stress has been identified as a cause for the “browning” (i.e., decrease in plant biomass) of Arctic tundra (Gonsamo et al., 2019; Myers‐Smith et al., 2020), and also for a decrease in the net growing‐season carbon uptake over the last decades (Angert et al., 2005; Wang et al., 2018). In addition, soil moisture affects Arctic plant species distribution (Bring et al., 2016; Kemppinen et al., 2021; Nabe‐Nielsen et al., 2017), and plant photosynthetic activity both directly (Dahl et al., 2017) and indirectly via influencing nutrient mineralization and absorption of nutrients by roots (Körner, 2003; López‐Blanco et al., 2020).

At the site level, the impacts of soil moisture on tundra CO_2_ fluxes are broadly understood. Drier conditions increase aerobic respiration resulting in a decrease in net carbon storage by tundra ecosystems, while wetter conditions conversely reduce soil decomposition and subsequent CO_2_ losses (Kwon et al., 2016; Lupascu et al., 2014; Oberbauer et al., 2007). Yet, a large‐scale analysis, which tested if soil moisture could explain a decrease in the correlation between the normalized difference vegetation index (NDVI) and air temperature in northern ecosystems, found an increase in soil moisture over the last decades, therefore suggesting that soil drying is not the main mechanism explaining the reduced response of plant productivity to temperature (Wang et al., 2018). However, the hydrology of northern high‐latitude ecosystems is complex, driven by the tight link between water drainage and the presence and depth of permafrost (Liljedahl et al., 2011). In addition, micro‐topography in Arctic ecosystems results in extreme plot‐scale variability in vegetation types, soil properties, and soil moisture content over the meter scale (Davidson et al., 2016; Wilkman et al., 2018; Zona et al., 2011), adding to the challenges of using coarser‐scale remote sensing products (e.g., from Landsat [30 m] or MODIS [250–1000 m]) to characterize the soil environment in these northern ecosystems. Satellite microwave soil moisture retrievals from the Soil Moisture Active Passive mission, purported to be one of the most reliable global soil moisture products (Zwieback et al., 2019), have a very coarse sampling footprint (~40‐km) and showed no meaningful correlation with in‐situ soil moisture in several Arctic ecosystems (Wrona et al., 2017). The disagreement between coarse soil moisture remote sensing products and the site‐level measurements is exacerbated by the sparsity of ground‐based data to estimate soil moisture across scales in Arctic ecosystems (Wrona et al., 2017). The lack of consistent long‐term data (i.e., more than 10 years) on site‐level plant productivity, carbon fluxes (e.g., eddy covariance), and soil moisture across northern ecosystems has limited our ability to directly reconstruct the temporal changes in the Arctic carbon balance resulting from environmental changes over recent decades. Given the complexity of these systems, the response of the tundra carbon exchange to moisture changes remains a key research question that requires evaluation across the Arctic (De Vrese et al., 2022; Göckede et al., 2019).

In this study, to capture the response of surface‐atmosphere fluxes of carbon dioxide to temperature and moisture at different stages of vegetation development, we investigate the correlation between air temperature (expressed as growing degree days, GDD), vapor pressure deficit (VPD), volumetric soil moisture content, net ecosystem exchange (NEE), gross primary production (GPP), and ecosystem respiration (ER) at different times of the summer. For this analysis, we used 11 eddy covariance sites distributed throughout the pan‐Arctic region. Given that different vegetation communities have different soil moisture optima for soil respiration and photosynthesis, we also tested the response of NEE, GPP, and ER by grouping sites based on their main vegetation classes. In fact, net CO_2_ uptake has been found to respond differently to increased temperature in wetter fens and drier tundra ecosystems (Grant et al., 2015). We expect higher GDD to be related to higher GPP, and greater net carbon uptake across these temperature‐limited ecosystems in all the summer months. This relationship would be especially present in the sites with more drought‐tolerant vegetation (e.g., shrub‐dominated ecosystems). We also expect soil moisture and VPD to limit plant productivity and carbon sequestration in July and August in the ecosystems dominated by graminoids and mosses, consistent with the progressive soil and moss drying during the summer. Finally, we expect a steeper response of ER to increases in GDD in dryer shrub dominated ecosystems given that higher soil aeration should stimulate ER and decomposition rates to a higher degree.

## 2 METHODS

### 2.1 Study sites

**FIGURE 1:**
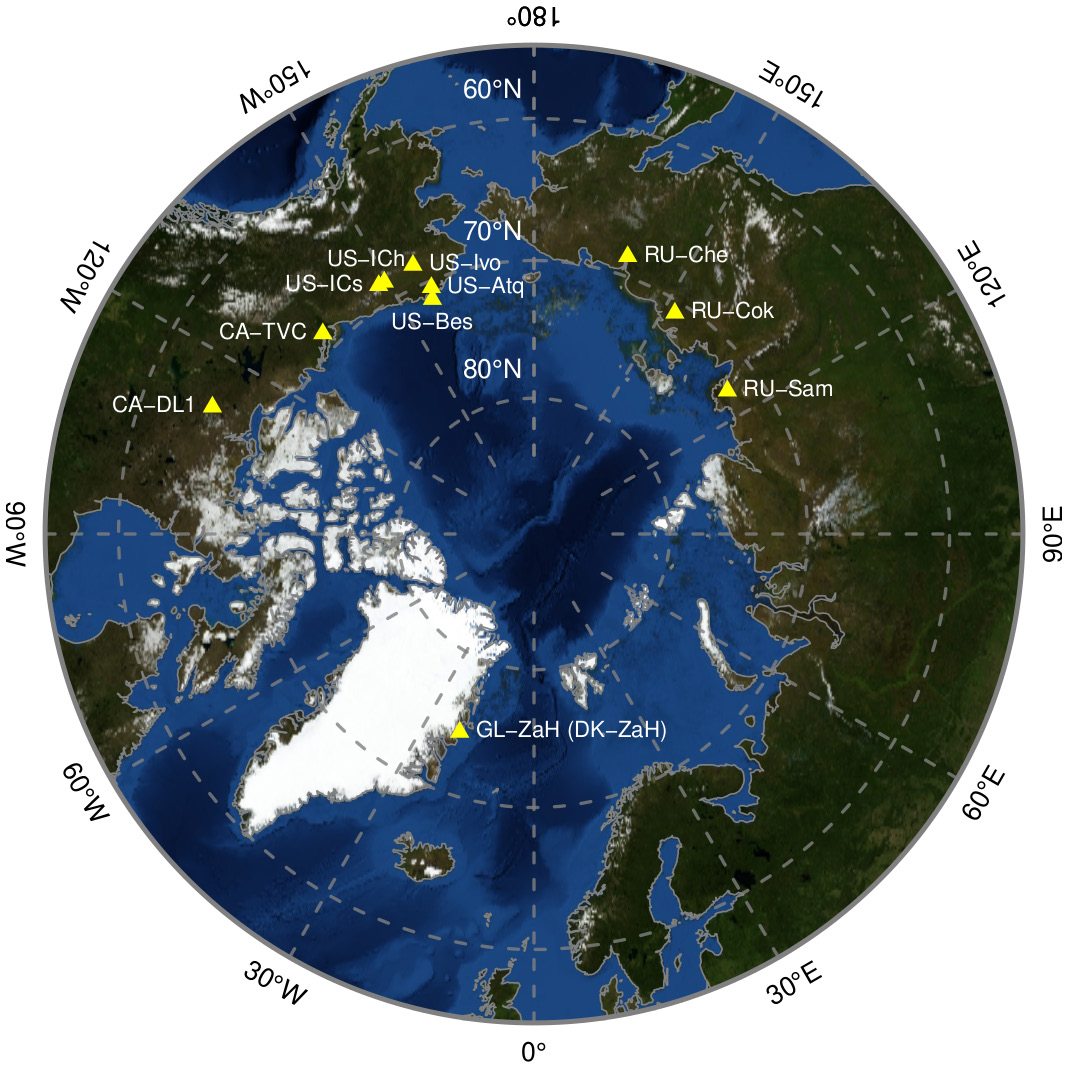
Location of the 11 eddy covariance (EC) flux tower sites included in this study. All sites are located over continuous permafrost. Details on the vegetation types, the available time periods, the average summer soil moisture at each site, and references describing the sites are included in Table 1.

A total of 11 eddy covariance sites distributed throughout the pan‐Arctic region were used in this study (Figure 1). The dataset comprises 99 site‐years of data including NEE, GPP, ER, and GDD; 74 site‐years included soil moisture data. Some sites had no soil moisture available for some years, and RU‐CoK had no soil moisture available for the entire time period, Table 1. This dataset included a time range from 6 to 19 years for each site (Table 1). All sites are located in continuous permafrost tundra regions. The 11 sites encompassed a range of tundra types, as classified by Walker et al. (2005); see Table 1, and moisture status. The wet end of this continuum (with soil moisture more than 60%, see Table 1) included sedge/grass, moss and shrub wetlands, the intermediate moisture level included graminoid and tussock tundra and had an average soil moisture around 47%–60% (Table 1), and dwarf and erect shrub tundra occupied the drier end with soil moisture about 42%–31% (Table 1). The average summer (June–August) GDD, and soil moisture are included in Table 1. GDD was estimated as the sum of the mean daily air temperatures above 5°C for the entire summer, and for each of the three summer months (June, July, and August) separately (Ueyama et al., 2013).

**TABLE 1:** Summary of eddy covariance data used in this study. Indicated are the locations, the years for which data for each of the sites are included in this study, the vegetation type classification according to Walker et al. (2005), the average summer soil moisture and GDD, and their 95% CI (%), and the main references describing the site. Modified from Zona et al. (2022)

AmeriFlux SITE‐ID	Vegetation	Coordinates	Flux years	GDD June‐August (°C)	Soil moist June‐August (%)	References
US‐Bes	W1 sedge/grass moss wetland	71.281 N, 156.596 W	2005–2011 2014–2019	64 ± 23	62 ± 1	Goodrich et al. (2016); Zona et al. (2016)
US‐Atq	W2 sedge moss/dwarf‐shrub wetland	70.470 N, 157.409 W	2004–2008 2011–2019	292 ± 48	53 ± 5	Goodrich et al. (2016); Zona et al. (2016)
US‐Ivo	G4 tussock‐sedge, dwarf‐shrub, moss tundra	68.49 N, 155.750 W (2004–2007) 68.481 N, 155.757 W (2014‐)	2004–2007 2013–2018	419 ± 80	60 ± 7	Zona et al. (2016); Goodrich et al. (2016)
US‐ICh	G4 tussock‐sedge, dwarf‐shrub, moss tundra	68.607 N, 149.296 W	2008–2019	346 ± 41	66 ± 3	Euskirchen et al. (2006); Euskirchen et al. (2017); Kade et al. (2012)
US‐ICs	G4 tussock‐sedge, dwarf‐shrub, moss tundra	68.606 N, 149.311 W	2008–2019	362 ± 40	64 ± 5	Euskirchen et al. (2006); Euskirchen et al. (2017); Kade et al. (2012)
GL‐ZaH (DK‐ZaH)	P2 prostrate/hemiprostrate dwarf‐shrub tundra	74.473 N, 20.550 W	2000–2019	124 ± 25	31 ± 5	Lund et al. (2012)
RU‐Che	W3 sedge, moss, low‐shrub wetland	(2003–05): N 68.613 E 161.341 (2013‐): N 68,617 E 161.351	2003–2004 2013–2016 (April‐Nov)	642 ± 25	47 ± 2	Göckede et al. (2019); Kwon et al. (2019)
RU‐Cok	W2 sedge moss/dwarf‐shrub wetland	70.830 N, 147.489 E	2003–2013 (n/a)	323 ± 42	n/a	Parmentier et al. (2011)
RU‐Sam	W2 sedge moss/dwarf‐shrub wetland	72.373 N 126.498 E	2008–2010 2013–2017	310 ± 84	51 ± 4	Boike et al. (2013) Boike et al. (2019); Holl et al. (2019); Sachs et al. (2010)
CA‐DL1	S1 erect dwarf‐shrub tundra	64.869 N, 111.575 W	2004–2019	579 ± 54	35 ± 2	Humphreys and Lafleur (2011); Lafleur and Humphreys (2008)
CA‐TVC	S1 erect dwarf‐shrub tundra	68.746 N, 133.502 W	2013–2019	568 ± 43	42 ± 2	Helbig et al. (2016)

Based on vegetation characteristics described in Walker et al. (2005), we arranged the sites into two separate groups: (1) the “graminoid and moss dominated” which included US‐Bes, US‐Atq, RU‐Sam, RU‐Cok, RU‐Che, US‐Ivo and US‐ICs, and (2) the “shrub‐dominated” ecosystems which included GL‐ZaH, CA‐DL1, CA‐TVC, and US‐ICh (Table 1). The US‐ICh was included in the “shrub‐dominated” group after discussion with the site PI, as it is a dry heath tundra ecosystem dominated by *Dryas integrifolia*, lichen, *Carex spp*., dwarf evergreen, and deciduous shrubs (Euskirchen et al., 2017). These groupings were based on the dominant vegetation at each of the sites: the sites classified as W1, W2, W3, and G4 by Walker et al. (2005) are mostly dominated by mosses, sedges, and grasses with only sparse or no shrub cover and have generally higher soil moisture, while the sites classified as P2 and S1 are dominated by shrubs and have generally lower soil moisture (Table 1).

### 2.2 Eddy covariance data processing and meteorological data

A full description of the eddy covariance data processing and details of the site level instruments are included in the references listed in Table 1. Tundra sites are appropriate for eddy covariance methods because they fulfill the assumption of flat terrain which is often violated in other ecosystems. Moreover, given the short stature of tundra vegetation, the eddy covariance instrumentation is only a few meters from the surface; footprint analyses from some of the sites included in this study showed an average fetch of about 200 m (Reuss‐Schmidt et al., 2019). Negative NEE indicates CO_2_ uptake by the ecosystem, and a positive NEE shows CO_2_ loss to the atmosphere. Missing data were gap‐filled according to the standard methodology of Ameriflux/Fluxnet for all sites (Pastorello et al., 2020), except for some years in US‐Bes, and US‐Atq due to large gaps where a neural network approach was employed as described in Goodrich et al. (2016). ER and GPP were estimated according to Lasslop et al. (2010) using the “REddyproc” package in R (Wutzler et al., 2018), as nighttime data are unavailable for most of the Arctic summer. The algorithm developed by Lasslop et al. (2010) partitions NEE using a hyperbolic light response curve to model GPP in combination with an exponential model term, to account for the temperature sensitivity of respiration. Additionally, the VPD limitation of photosynthesis is considered. The soil moisture data used in the study were measured at each site (except for RU‐Cok) using time‐domain reflectometry probes inserted in the moss or soil layers in proximity of the eddy covariance towers. We selected a subset of the available soil moisture sensors at each site to use consistent depths across sites, and sensors with the most complete record within each site (0–20 cm depth, Data S1). Air temperature and relative humidity were measured with an HMP45 Vaisala (Vaisala, Vantaa, Finland) and these data were used to calculate actual and saturation vapor pressures. VPD was calculated by subtracting the actual vapor pressure of the air from the saturated vapor pressure.

### 2.3 Statistical analysis

**FIGURE 2:**
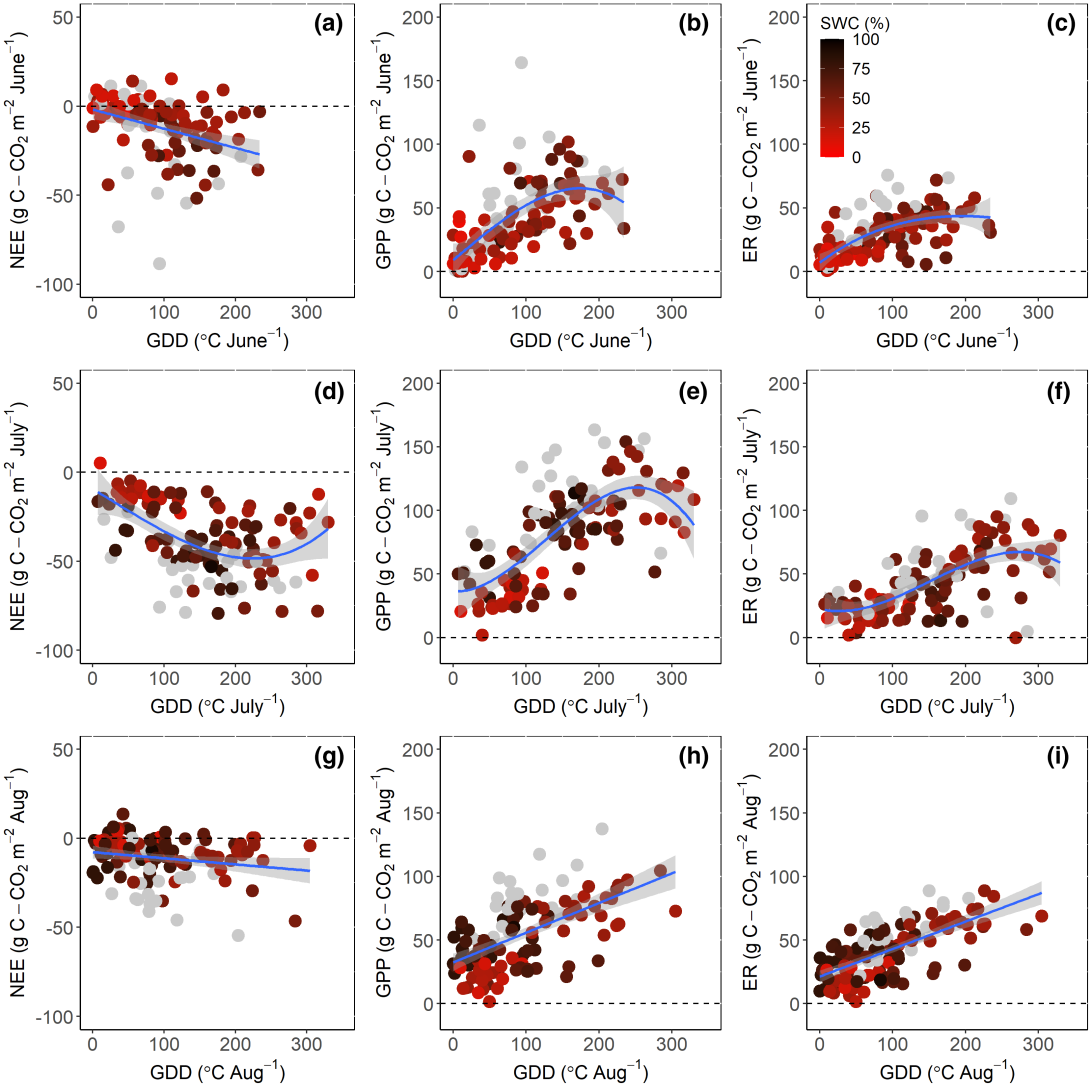
Relationships between the (a,d,g) monthly cumulative net ecosystem exchange (NEE), (b,e,h) gross primary productivity (GPP), (c,f,i) ecosystem respiration (ER), and growing degree days (GDD), for the months of June, July, and August, for the indicated moisture levels (grey dots indicates sites/years with no soil moisture available). Linear models were used when a polynomial fit was not significantly better as estimated by an ANOVA between the two models. The statistics of these relationships are included in Table 3. Negative values in NEE indicate a CO_2_ uptake by the ecosystems.

The relationship between VPD, soil moisture, and GDD for three different stages of the summer (early summer: June, peak summer: July, and late summer: August) was assessed using regressions and mixed‐effect models including site as random effect (Bates, 2010). These analyses were carried out for the three summer periods, given that shifting dynamics and phenological development of vegetation might mask the impact of temperature and soil moisture when the carbon balance is modeled for the entire summer season. The inclusion of “site” as a random effect in the mixed models allowed us to test the consistency between the results once accounting for the site‐to‐site variability and prevented potential artifacts arising from pseudo‐replication. To evaluate the sensitivity of NEE, GPP, and ER to GDD, we employed both linear and third‐degree polynomial models. A third‐degree polynomial model was used as it is more flexible than a second‐degree polynomial model and able to capture more complex relationships. Linear and polynomial models were compared to evaluate the nature of the relationship between GDD and NEE, GPP, and ER and to identify a potential decrease in GPP at the highest GDD. This comparison was accomplished by an analysis of variance (ANOVA) and a Chi‐square test. In the presentation of results, the polynomial model was used only when it statistically explained more of the variation than the linear model (Figure 2, Table 3). We also tested the performance of a mixed effect model including “year” of measurement, and “site” as continuous and categorical random effects, respectively, to account for the different sites measured in different years potentially affecting the NEE, GPP, ER, and GDD relationships, and to account for the non‐independence of the dataset (multiple points from the same sites in different years). Model performance was evaluated based on the Akaike information criterion, on the marginal coefficient of determination (similar to the explanatory power of the linear models) for generalized mixed effects models as output by the “r.squaredGLMM” function within the “MuMIn” package in R (Johnson, 2014; Nakagawa & Schielzeth, 2013). As the model performance did not significantly improve when including “year,” only “site” was included as a random effect in the mixed models (Tables 2, 3, 4, 5). To evaluate the model performance, we also examined the standardized residuals using the plot(model) function in R to evaluate that the assumption of normally distributed model residuals was met. All analyses were carried out in R version 4.2.0 (R Core Team, 2022).

**TABLE 2:** Pearson correlation coefficients (*r*) and coefficient of determination (*R* ^2^) for the linear model, and *R* ^2^_m_ for the mixed effect model (including site as a random effect) for the relationships between the monthly growing degree day (GDD), the average monthly vapor pressure deficit (VPD), the average monthly soil moisture, for the indicated months

		GDD	
	June	July	August
VPD	*r* = .91	*r* = .87	*r* = .73
	*R* ^2^ = .83	*R* ^2^ = .77	*R* ^2^ = .52
	*p* < .001	*p* < .001	*p* < .001
	*R* ^2^_m_ = .72	*R* ^2^_m_ = .77	*R* ^2^_m_ = .52
	*p* < .001	*p* < .001	*p* < .001
Soil moisture	*r* = .36	n.s.	*r* = −.35
	*R* ^2^ = .13		*R* ^2^ = .12
	*p* = .0018		*p* = .0020
	*R* ^2^_m_ = .12	n.s.	n.s.
	*p* = .011		

**TABLE 3:** Statistics of the relationship between the cumulative monthly GPP, NEE, and ER and GDD for all sites using three different models: Linear model (Lm), a third‐degree polynomial model (Poly), and a mixed‐effect model including site as random effect (Mem). An asterisk is included (*) if the polynomial model was significantly different (*p* < .05) than a linear model as assessed from an ANOVA and a Chi‐square test between the two models

		GDD	
	June	July	August
NEE	Lm: *R* ^2^ = .11 *p* < .001	Lm: *R* ^2^ = .12 *p* < .001	Lm: *R* ^2^ = .07 *p* = .0082
	Poly: *R* ^2^ = .14 *p* = .003	Poly*: *R* ^2^ = .27 *p* < .001	Poly: *R* ^2^ = .11 *p* = .010
	Mem: *R* ^2^_m_ = .18 *p* < .001	Mem: n.s.	Mem: *R* ^2^_m_ = .062 *p* = .019
GPP	Lm: *R* ^2^ = .31 *p* < .001	Lm: *R* ^2^ = .40 *p* < .001	Lm: *R* ^2^ = .36 *p* < .001
	Poly*: *R* ^2^ = .37 *p* < .001	Poly*: *R* ^2^ = .49 *p* < .001	Poly: *R* ^2^ = .38 *p* < .001
	Mem: *R* ^2^_m_ = .33 *p* < .001	Mem: *R* ^2^_m_ = .11 *p* < .001	Mem: *R* ^2^_m_ = .26 *p* < .001
ER	Lm: *R* ^2^ = .35 *p* < .001	Lm: *R* ^2^ = .45 *p* < .001	Lm: *R* ^2^ = .46 *p* < .001
	Poly*: *R* ^2^ = .41 *p* < .001	Poly: *R* ^2^ = .47 *p* < .001	Poly: *R* ^2^ = .48 *p* < .001
	Mem: *R* ^2^_m_ = .32 *p* < .001	Mem: *R* ^2^_m_ = .31 *p* < .001	Mem: *R* ^2^_m_ = .32 *p* < .001

Abbreviations: ER, ecosystem respiration; GDD, growing degree days; GPP, gross primary productivity; NEE, net ecosystem exchange.

**TABLE 4:** Statistics of the interaction term between GDD and vegetation type (a linear model was used if not significantly different from polynomial or polynomial if significantly better than linear for a *p* ≤ .05); the *p*‐values of the interaction term between vegetation type and GDD were estimated for each of the months separately for the indicated models (Lm‐linear mode, Poly: third‐order polynomial), and for the mixed‐effect models (Mem) which included site as random effect

		GDD	
	June	July	August
NEE	Vegetation type*GDD: Lm: *p* = .73	Vegetation type*GDD: Poly: *p* = .081	Vegetation type*GDD: Lm: *p* = .088
	Mem: *p* = .74	Mem: *p* = .73	Mem: *p* = .16
GPP	Vegetation type*GDD: Poly: *p* = .015	Vegetation type*GDD: Poly: *p* = .92	Vegetation type*GDD: Lm: *p* = .33
	Mem: *p* = .64	Mem: *p* = .73	Mem: *p* = .24
ER	Vegetation type*GDD: Poly: *p* < .001	Vegetation type*GDD: Lm: *p* = .038	Vegetation type*GDD: Lm: *p* = .047
	Mem: *p* = .12	Mem: *p* = .90	Mem: *p* = .86

Abbreviations: ER, ecosystem respiration; GDD, growing degree days; GPP, gross primary productivity; NEE, net ecosystem exchange.

**TABLE 5:** Statistics of the partial correlation analysis between the indicated variables statistically accounting for GDD, using the model selected based on the results of Tables 3 and 4 (i.e., a polynomial model only if significantly better than a linear one, and including vegetation type only if the interaction term of GDD*vegetation type was significant for a *p* ≤ .05) to generated the residuals in GPP, NEE, and ER then used in the partial correlations between the indicated variables. Included are the *R* ^2^ of the linear model of the residuals in the NEE, GPP, and ER and soil moisture controlling for GDD, and a mixed‐effect model *R* ^2^_m_ including site as a random effect to account for the site‐to‐site variability

	Soil moisture (controlling for GDD)		
	June	July	August
NEE (controlling for GDD)	*r =* −.40	*r =* −.54	*r =* −.14
	*R* ^2^ = .16	*R* ^2^ = .29	*R* ^2^ = .02
	*p* < .001	*p* < .001	*p* = .22
	*R* ^2^_m_ = .12	*R* ^2^_m_ = .13	*R* ^2^_m_ = 0
	*p* = .012	*p* = .023	*p* = 0
GPP (controlling for GDD)	*r =* .31	*r =* .47	*r =* .34
	*R* ^2^ = .10	*R* ^2^ = .22	*R* ^2^ = .12
	*p* = .0068	*p* < .001	*p* = .0031
	*R* ^2^_m_ = .039	*R* ^2^_m_ = .062	*R* ^2^_m_ = .0089
	*p* = .0024	*p* = .094	*p* = .57
ER (controlling for GDD)	*r =* .18	*r =* −.068	*r =* .17
	*R* ^2^ = .033	*R* ^2^ = .0046	*R* ^2^ = .029
	*p* = .12	*p* = .57	*p* = .15
	*R* ^2^_m_ = .023	*R* ^2^_m_ = .028	*R* ^2^_m_ = .004
	*p* = .19	*p* = .33	*p* = .71

Abbreviations: ER, ecosystem respiration; GDD, growing degree days; GPP, gross primary productivity; NEE, net ecosystem exchange.

To evaluate if average monthly VPD and soil moisture were associated with cumulative CO_2_ uptake (NEE), GPP, and ER for June, July, and August, we used a partial correlation analysis. Here the cumulative NEE, GPP, and ER were each modeled as a function of soil moisture and VPD considering GDD for each month separately. Partial correlation statistically eliminates the impact of other controlling climate variables, which allowed us to evaluate the impact of soil moisture on GPP, NEE, or ER without the confounding effect of temperature (i.e., GDD). We also performed a similar partial correlation analysis to test the relationships between the cumulative NEE, GPP, and ER, against soil moisture and VPD (statistically controlling for GDD) within a mixed effect model, including site as a random effect. We tested if the relationship between the monthly cumulative GPP, NEE, and ER with GDD, and the relationship between GPP, NEE, and ER and soil moisture (once accounted for GDD) were different between vegetation classes (“graminoid and moss dominated” and “shrub dominated”).

We also used partial correlation analysis to test if accounting for soil moisture modified the relationship between GPP (or NEE) and GDD in July. We regressed the residuals of GPP (and NEE) with the residuals of GDD after removing the impact of soil moisture, and then tested again if a polynomial model explained data variability better than a linear model of GPP and GDD residuals. For this analysis, we selected the model based on the criteria previously listed to generate the residuals used for Figure 4 and Table 5. Specifically, vegetation type was included in the model to test the relationships between the residuals in NEE, GPP, ER, and soil moisture after removing the impact of GDD as shown in Figure 4, only when the interaction term between vegetation type and GDD was significant in explaining the variability in NEE, GPP, or ER, and, as mentioned previously, a polynomial fit was only used when significantly better than a linear fit.

## 3 RESULTS

The sites and years included in this study spanned a wide range of soil moisture conditions and summer GDD (Table 1). The average summer (June–August) soil moisture ranged from 31 ± 5% (mean ± 95% CI) at GL‐ZaH to 66 ± 1% at US‐ICh. GDD in June–August ranged from 64 ± 23°C at US‐Bes to 642 ± 25°C at RU‐Che (Table 1). Across all the sites, GDD were significantly (and strongly) positively correlated with VPD (i.e., higher GDD produced higher average VPD) in all summer months in both linear regression and mixed‐effects models (Table 2). GDD was significantly positively related to soil moisture in June, but not in July, and weakly negatively correlated to soil moisture in August. The results of the linear and mixed‐effects model were similar for June and July, but in August the mixed model did not show a significant relationship between soil moisture and GDD (Table 2). The driest and coldest conditions were associated with the lowest GPP, lowest net CO_2_ uptake, and ER fluxes, as shown by the lighter red points in Figure 2.

**FIGURE 3:**
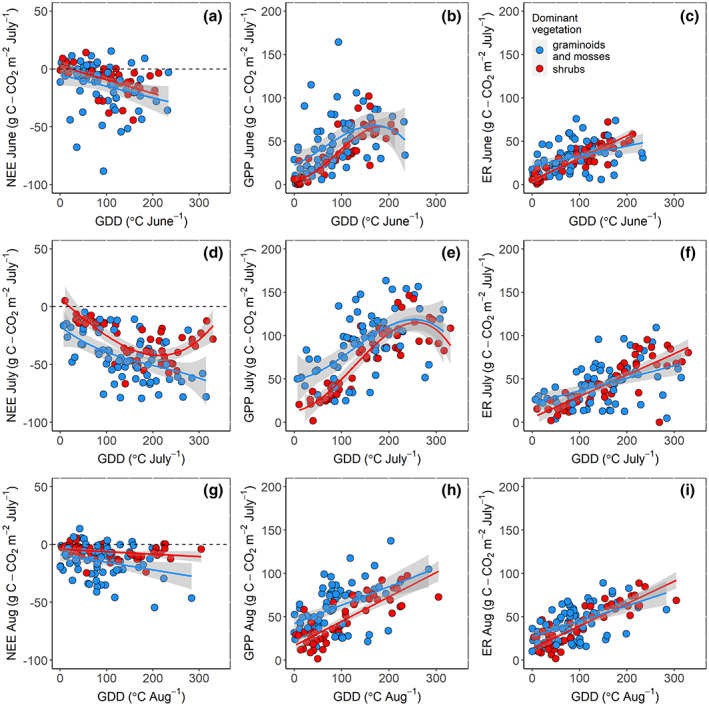
Relationships between the (a,d,g) monthly cumulative net ecosystem exchange (NEE), (b,e,h) gross primary productivity (GPP), (c,f,i) ecosystem respiration (ER), and growing degree days (GDD), for the months of June, July, and August, for the two indicated ecosystem types. The significance in the interaction term between GDD and vegetation type for each of the months and flux component is included in Table 4.

GDD was significantly related to NEE, GPP and ER in all months, but the percent of explained variance and the best model selected varied. Relationships with GDD were poorer for NEE than for GPP and ER. Polynomial relationships outperformed linear models for GPP and ER in June, and for NEE and GPP in July. In late summer (August), polynomial models were not significantly better than the linear models for any flux. The interaction term between vegetation type and GDD was only significant in explaining the variability in GPP in June, not significant in explaining the variability in NEE in any of the summer months, and always significant in explaining the variability in ER (in June–August, Table 4). When site was included as a random effect in the mixed‐effects models (Figure S1), the interaction term between vegetation type and GDD was never significant (Table 4). GPP and ER tended to increase with higher temperatures (i.e., GDD), and shrub ecosystems showed a steeper increase with an increase in GDD than moss and graminoid‐dominated ecosystems during all the summer months (Figure 3g–i, Table 4). GPP plateaued or slightly declined when GDD exceeded ~175°C and 250°C in June and July, respectively, (Figure 3b,e), and this decrease was consistent in both the moss and graminoids‐dominated and shrub‐dominated ecosystems in July (Figure 3e).

**FIGURE 4:**
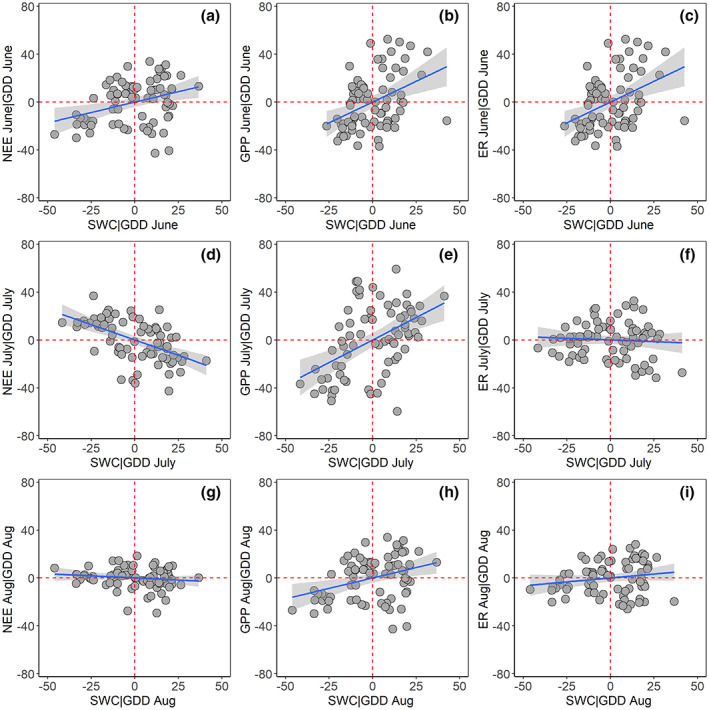
Partial correlation between the (a,d,g) monthly cumulative net ecosystem exchange (NEE), (b,e,h) gross primary productivity (GPP), (c,f,i) ecosystem respiration (ER), and the average monthly soil moisture, while statistically controlling for growing degree days (GDD) for the months of June, July, and August. The interaction term between vegetation type and GDD was included in the partial correlation model to generate the residual displayed here only when significant as reported in Table 4. The statistics of the relationships displayed in these panels are presented in Table 5.

The partial correlation analysis showed that when statistically controlling for GDD, VPD was not significantly related to GPP, NEE, and ER in any of the summer months (data not shown). The coefficients of the partial correlations between GPP and soil moisture (accounting for GDD) were positive in all summer months (i.e., higher plant productivity with higher soil moisture, Table 5). The correlation between NEE and soil moisture was significant (and negative) in June and July (i.e., more net CO_2_ uptake with higher soil moisture), but not significant in August (Table 5). The partial correlation between ER and soil moisture was not significant in any of the summer months for both the linear and mixed effect models (Table 5). The explanatory power (*R* ^2^) of the linear and mixed effect models testing the relationships between GPP (or NEE) and soil moisture were low (less than 30%), with generally lower explanatory power for the mixed‐effect models compared to the linear models (Table 5). After removing the influence of soil moisture on the GPP vs GDD relationship (e.g., by regressing the residuals of both the correlation between GPP and soil moisture, and GDD and soil moisture), there was only a marginally significant difference between the polynomial and linear models explaining the relationship between the residuals of GPP and the residuals of GDD in the partial correlation analysis (*p* = .054). Additionally, there was no significant difference between the polynomial and linear models in explaining the relationship between the residuals of the NEE and soil moisture relationship and the residuals of GDD and soil moisture in the partial correlation analysis (*p* = .55).

## 4 DISCUSSION

As expected, VPD and GDD were strongly correlated in all summer months, given that atmospheric warming and drying are associated with each other through the non‐linear increase in saturation vapor pressure with air temperature. The relationship between GDD and soil moisture was more complex. The positive correlation between GDD and soil moisture in June (i.e., wetter soils with warmer conditions) was likely due to the progressive snow melt and soil thawing at the beginning of the summer. This interpretation is supported by the significant relationship between the end of the snow melt period and soil moisture only in June, and not significant in July and August (Zona et al., 2022), when the active layer approaches its maximum depth. The non‐significant relationship between GDD and soil moisture in July and August in the mixed effect model suggests the lack of a consistent response of the peak and late season soil moisture to GDD among sites. Soil drying is limited by the increase in bulk surface resistance to evapotranspiration with increased VPD‐driven (increasing with higher temperatures) stomatal closure (Grant et al., 2015). The drying of the moss surface in peak summer in wet tundra further limits the ability to transfer moisture from the soil (Liljedahl et al., 2011). Overall, the impact of soil moisture on both GPP, and NEE was the highest during the peak season, and is consistent with a decrease in GPP, and net carbon uptake at the highest GDD in July. This potential peak summer soil moisture limitation is supported by the better performance of a polynomial than a linear fit explaining the relationship between GDD and both GPP and NEE in July. The polynomial fit was able to capture the increase in GPP with an increase in GDD until about 250°C for July, followed by the decrease in GPP at the highest GDD. Although including site as a random effect decreased the explanatory power of the model (as shown by the lower $R_{m}^{2}$ in July), the model retained its significance. The increase in GPP with GDD until ~250°C suggests that a moderate warming is beneficial to tundra plants. However, a higher degree of warming might negatively affect the tundra vegetation as shown by the higher performance of the polynomial fit in explaining the relationship between GPP and GDD in July. The decrease in plant productivity at the sites subjected to the warmest conditions was also observed in the mixed effect model, once accounting for site‐to‐site variability. This result is consistent with a non‐linear response of photosynthesis to temperature in Arctic ecosystems (Ackerman et al., 2017; Piao et al., 2014). In particular, only sites exposed to the highest GDD (i.e., GDD higher than 250°C) showed a plateau (or a negative slope) in the GPP and GDD relationship (Figure S1).

The similar decrease in GPP with GDD above ~250°C in both moss and graminoid‐dominated and shrub‐dominated ecosystems in July was surprising. We expected GPP to decrease at the highest GDD only in the ecosystems dominated by mosses. In fact, desiccation associated with warming limits photosynthetic CO_2_ uptake in poikilohydric plants, which includes mosses (Oberbauer et al., 2007; Van Wijk et al., 2004; Yang et al., 2018), given their lack of rooting system. Warming has been generally associated with a reduction in biomass accumulation in mosses, but with an increase in shrubs (Bao et al., 2022). The rooting system of shrubs allows them to access water in deeper soil layers making them physiologically better adapted to drier soils (Cahoon et al., 2016). However, a higher intensity of warming (by 1.51°C) has been shown to enhance the abundance of shrubs and graminoids by half as much (10% vs. 20%) than a lower warming intensity (by 1.04°C) (Bao et al., 2022) suggesting that moisture stress can also affect vascular plants' productivity. Decreases in soil moisture have been found to limit shrub expansion and growth (Boulanger‐Lapointe et al., 2014; Naito & Cairns, 2011). Moreover, both leaf area and leaf nitrogen content have been found to decrease with temperature in drier tundra ecosystems (Bjorkman et al., 2018; Grant et al., 2015). Shrub growth is more sensitive to temperature in wetter vs. drier sites (Ackerman et al., 2017; Myers‐Smith et al., 2015). Moisture limitation can decrease shrub growth, recruitment, and abundance (Ackerman et al., 2017; Elmendorf et al., 2012; Li et al., 2016; Myers‐Smith et al., 2015). Our results suggest that even if physiologically better adapted to drier soils, shrub‐dominated ecosystems can experience a limitation in their photosynthetic carbon uptake with warming and drying, which is most evident during peak season.

Confirming our last prediction, the increase in ER with increase in GDD was always steeper in shrub‐dominated ecosystems, consistent with the higher sensitivity of drier ecosystems to warming (Hodkinson et al., 1999; Oberbauer et al., 2007). The higher soil aeration in the drier shrub‐dominated ecosystems stimulates ER and decomposition (Grant et al., 2015; Oberbauer et al., 2007). Shrub‐dominated ecosystems showed a lower ER at lower GDD, consistent with the less pronounced effects of moisture on metabolism at lower temperatures (Fischer, 1995; Fischer & Bienkowski, 1987), and possibly related to the lower plant productivity and lower plant biomass under drier and colder conditions. Overall, both tundra vegetation types remained a summer carbon sink because GPP increased more than respiration with GDD, opposite to what Parmentier et al. (2011) found.

The importance of soil moisture for the response of tundra ecosystems to warming is consistent with the dominant role of water availability on plant growth across different tundra ecosystem types (Bjorkman et al., 2018; Hodkinson et al., 1999). The significant relationships between soil moisture and both NEE and GPP in the partial correlation analysis (after controlling for GDD) and in the mixed effect model in June and July emerged from different ecosystem processes occurring in the early and peak summer. In June, soil moisture is low mostly due to soil water still being frozen, as previously described. Warmer conditions, co‐occurring with increased soil moisture, activate the vegetation, increasing GPP, and net CO_2_ uptake. The significant partial correlations of soil moisture with GPP (or NEE) in the peak summer suggest that the decrease in GPP at the highest GDD might be due to soil moisture limitation in July. The potential role of soil water limitation in explaining the plateau (or slight decrease) in the GPP and GDD relationship at the highest GDD is supported by the partial correlation analysis. After removing the impact of soil moisture, the polynomial model (which can capture the plateau in the GPP/GDD relationship) was only marginally significantly better than a linear model. A similar result was observed for NEE. Once removing the impact of soil moisture, the polynomial model was not significantly better than a linear model in explaining the relationship between the residuals of NEE and GDD. Overall, these results suggest that soil moisture limits the increase in GPP (and the net CO_2_ uptake) at the highest GDD across very different vegetation types. Beyond the site scale, drier conditions have been associated with the lack of a positive trend in the minimum late summer atmospheric [CO_2_] (a proxy of photosynthetic CO_2_ uptake) in northern ecosystems since the 1990 s (Angert et al., 2005). This finding is consistent with the observed decrease in the net CO_2_ uptake with drainage in other tundra ecosystems (Kittler et al., 2017; Pegoraro et al., 2021). Snow melt date and soil moisture in July are not correlated (Zona et al., 2022), suggesting that the peak summer soil moisture was not substantially influenced by the timing of seasonal soil thawing.

In late summer, soil moisture was generally not significant in explaining NEE, and GPP after accounting for GDD in the partial correlation analysis, as the onset of senescence might decrease the water demand from vegetation. At this time of the year, other environmental factors, such as temperature and light, may become more important than soil moisture in limiting plant growth in these northern ecosystems (Starr et al., 2008). We found that in August the explained variance for the relationship between GDD and GPP (or NEE) was similar between the polynomial model and the linear model, suggesting that soil moisture did not limit plant productivity and net CO_2_ sequestration during this time of year. Some studies have suggested that warmer conditions do not affect plant growth during the late season as the photoperiod is the dominant control on phenology at the end of the growing season in the high‐Arctic (e.g., Arft et al., 1999), while others show the opposite (e.g., Kittler et al., 2017) and suggest that warmer temperatures can delay plant senescence (Marchand et al., 2004; Zeng et al., 2011). Our previous analysis across these same sites (Zona et al., 2022) showed that air temperature was generally less important in August than in June and July, while solar radiation retained similar importance in explaining the variability in GPP during all the stages of the growing season. Importantly, GDD was rarely above 250°C in August so that excess heat and associated moisture limitation were not likely driving GPP down, consistent with the similar explanatory power of the polynomial and linear models in late season.

The non‐significant partial correlations between NEE and VPD and between GPP and VPD (after accounting for GDD) suggest a lack of atmospheric moisture limitation on plant productivity and net CO_2_ uptake across these tundra ecosystems. These results are consistent with a similar partial correlation analysis by Wang et al. (2018), testing the temporal changes of NDVI and showing a positive or non‐significant partial correlation with VPD when the data were statistically controlled for the effect of mean summer temperature. On the other hand, stomatal closure of vascular plants at high temperature and VPD has been shown to limit tundra carbon uptake (Grant et al., 2015; Williams et al., 2000). The lack of a significant association between VPD and GPP, or NEE in the partial correlation analysis might be the result of the strong correlation between air temperature (i.e., GDD) and VPD. Once accounting for GDD in the partial correlation analysis, the residual variation in GPP or NEE was not significantly explained by VPD. Such challenges in ranking strongly co‐varying environmental controls in observational studies have been recognized before (Zona et al., 2009).

Most ecosystem models do not capture the recent decrease in the sensitivity of plant productivity to temperature in northern ecosystems (Piao et al., 2014; Wang et al., 2018). The non‐linear responses of NEE and GPP to GDD found in our study reinforce the hypothesis that northern tundra ecosystems' responses to temperature can be limited by reduced soil moisture. It has been suggested that the role of soil moisture has been underestimated in the Arctic and alpine systems (le Roux et al., 2013). Our results and others (Angert et al., 2005; Feng et al., 2021; Gonsamo et al., 2019) support this view. Therefore, soil moisture limitation to plant growth and net carbon sequestration should be given greater consideration when modeling the response of northern ecosystems to global warming. This is critical when projecting future tundra greening/browning trends: models must accurately incorporate the combined non‐linear effects of temperature and soil moisture on productivity to correctly capture the sign and magnitude of Arctic carbon balance. The sparsity of continuous flux data from these high‐latitude ecosystems was the main challenge we faced when performing our study and harmonizing the datasets, together with the challenges in partitioning the fluxes with large gaps in the meteorological datasets. The Arctic research community should attempt to include additional sites in a wider range of vegetation types over continuous permafrost (Pallandt et al., 2022), improve coverage of the soil moisture data, and extend the sampling to cold periods to improve an overall estimate of the year‐round response of ecosystems to warmer temperature.

## Supporting information


Data S1

## Data Availability

The eddy covariance data from RU‐Che, RU‐Cok, and GL‐ZaH (previously named DK‐ZaH), CA‐DL1, were obtained from the European Fluxes Database (http://www.europe‐fuxdata.eu/home), from the Amerifux Database (http://amerifux.lbl.gov/), with some updated versions provided directly by the principal investigators of each site (e.g., the data from GL‐ZaH are also available on: https://data.g‐e‐m.dk). The data from US‐ICh and;US‐ICs are stored in the http://aon.iab.uaf.edu/data_access. US‐Bes, US‐Atq, US‐Ivo are stored in the Arctic Data Center (Donatella Zona. 2022. Greenhouse gas flux measurements at the zero curtain, North Slope, Alaska, 2012‐2022. Arctic Data Center. doi:10.18739/A20Z70Z1H, version: urn:uuid:38b9ea29‐67ba‐4d52‐827d‐922cbb8e0168).
