# Supplementary material for: Pan‐Arctic soil moisture control on tundra carbon sequestration and plant productivity
Source: Glob Chang Biol. 2022 Nov 10;29(5):1267–81. doi: 10.1111/gcb.16487 (PMC10099953; doi:10.1111/gcb.16487)
Supplement: Supplementary file 1 — Data S1 [file GCB-29-1267-s001.docx]

**Supplementary Information**

**Soil moisture sensors across the study sites**

| Site | Years | Sensor number | Sensor location |
| --- | --- | --- | --- |
| CA-DL1 | 2004-2019 | N=2 | horizontal; one in a wet location and one in a dry location at -10 cm depth |
| US-Atq | 2010-2019 | N=1 | horizontal -10 cm depth |
| US-Ivo | 2014-2019 | N=4 | horizontal at -5 cm depth |
| US-Bes | 2006-2010  2012-2019 | N= 2 | diagonally inserted at 0-10cm |
| US-Che | 2003-2004  2013-2016 | N=2 | horizontal -8cm and -16cm depth |
| RU-Sam | 2009-2010  2013-2017 | N=5 | horizontal at -5, -14, in rim ,  -5, -12, -15 cm in the center of ice-wedge polygon |
| US-ICt | 2008-2019 | N=2 | horizontal at -2.5 cm |
| US-ICh | 2008-2019 | N=2 | horizontal at -2.5 cm |
| GL-ZaH | 2000-2004  2005- | N=2 | vertical 0-6 cm  horizontal: -5cm, -10 cm |
| CA-TVC |  | N=1 | horizontal -20cm |

The average soil moisture indicated in Table 1 in the main manuscript includes all the sensors available at the sites; the number of sensors and the soil depths in each of the sites are listed for each site (CA-DL1: N=2 (in a wet location and a dry location, both at -10 cm depth); US-Atq: N=4 (2010-2013, at -5 (2),-15, and -30 cm depth), N=12 (2014-2019 at -5 (5),-15 (4), and -30 cm (3) depth); US-Ivo: N= 12 (4 at -5 cm depth, 4 at -15 cm depth, and 4 and -30 cm depth); US-Bes: N= 5 (2 diagonally inserted at 0-10 cm, 1 diagonally inserted at -20-30 cm, 2 vertically inserted at 0-30 cm depth); US-Che: N=2 (-8cm and -16cm depth); RU-Sam: N=11 (4 in slopes at -5, -14, -23, -33 depth, and 7 in rims at -5, -12, -15, -22, -26,- 34, and -37 cm depth); US-ICt: N=2 (at -2.5 cm depth); DK-ZaH: N=2 (2000-2004 vertical 0-6 cm and from 2005 onward are at two depths horizontal: -5cm, -10 cm depth) CA-TVC: N=1 ( inserted horizontally at -20cm depth).

**Supplementary Figures**


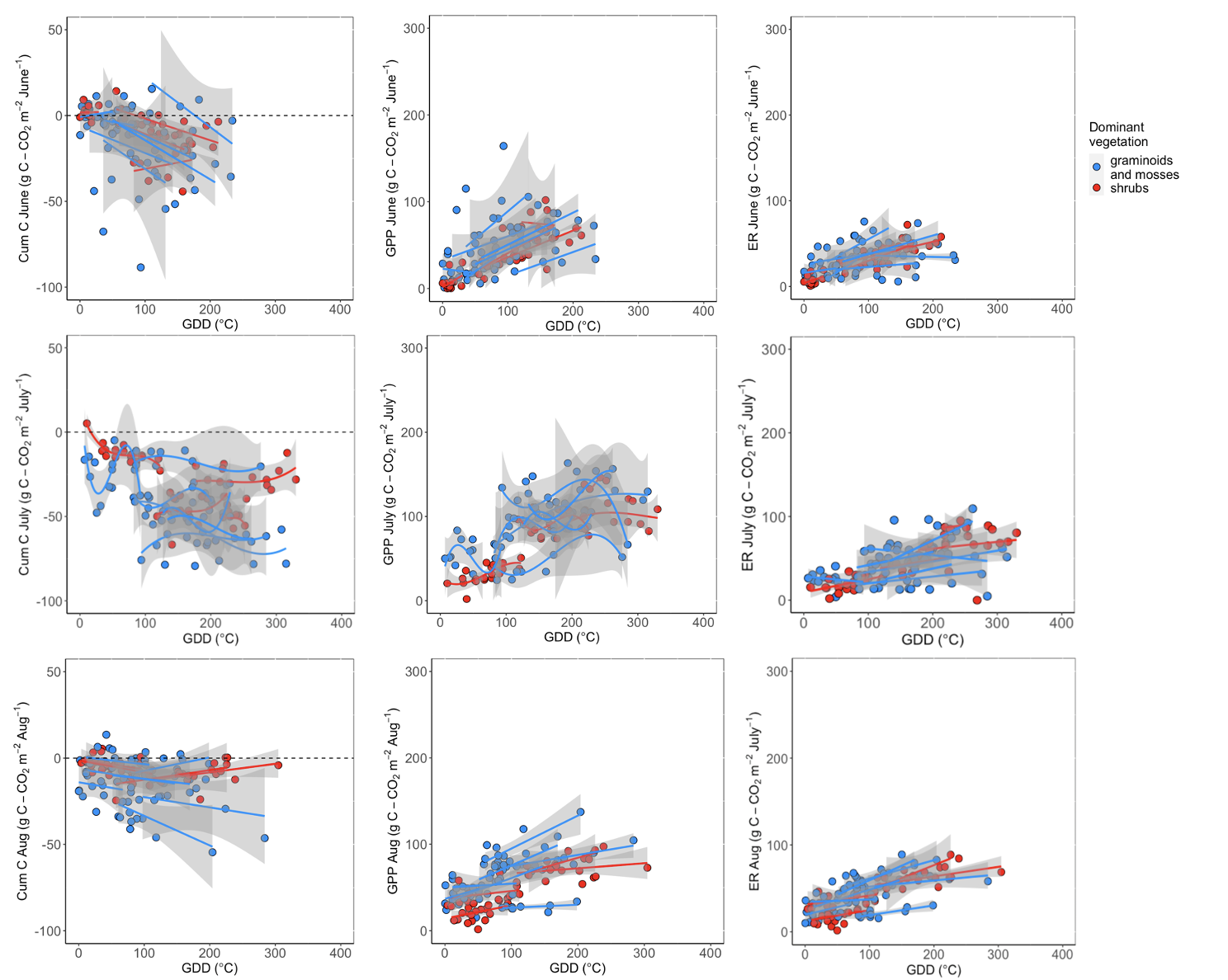


**Supplementary Fig. 1** Relationships between the monthly cumulative net ecosystem exchange (NEE), gross primary productivity (GPP), ecosystem respiration (ER) and growing degree days (GDD), for the month of June, July, and August, for the 11 sites (the regression lines are separate for each site); in the figure legend the sites are grouped into two main categories based on the dominant vegetation (as described in the Methods section).

**Funding support for this study**

Funding for E.E. was provided by the NSF Arctic Observatory Network (award numbers 0732594, 1107892, 1503912, and 1936752). LK and DH received funding from the Deutsche Forschungsgemeinschaft (DFG, German Research Foundation) under Germany‘s Excellence Strategy – EXC 2037 'CLICCS - Climate, Climatic Change, and Society' – Project Number: 390683824, contribution to the Center for Earth System Research and Sustainability (CEN) of Universität Hamburg. A portion of this work was performed at the Jet Propulsion Laboratory, California Institute of Technology, under contract with National Aeronautics and Space Administration (80NM0018D0004). A.K.L. acknowledges support from NSF-OPP 1722572. H.K. acknowledges support from the NSF Macrosystems Biology program (award EF-1065029) and the Belgian Science Policy Office (contract BR/175/A3/COBECORE). Funding for J.D.W. was provided through the NASA New Investigator Program (NNH17ZDA001N-NIP) and a grant through the Gordon and Betty Moore Foundation. G.B. acknowledges both LI-COR Biosciences and the University of Nebraska for allowing the time to work on the manuscript. X.X. acknowledges the financial support from the Department of Energy – Oak Ridge National Laboratory (4000145166). This work partially used the Extreme Science and Engineering Discovery Environment (XSEDE), which is supported by National Science Foundation grant number ACI-1053575. Data from Zackenberg was provided by the Greenland Ecosystem Monitoring (GEM) program. Data collection at Ru-Sam was supported by the Cluster of Excellence “CliSAP” (EXC177), University of Hamburg, funded by the German Research Foundation (DFG) and the Helmholtz Association of German Research Centres (grant VH-NG-821 to T.S.) and the Helmholtz infrastructure funding ACROSS (Advanced Remote Sensing - Ground Truth Demo and Test Facilities). The work at Trail Valley Creek was funded through the Canada Research Chairs Program, the Canada Foundation for Innovation, the Polar Continental Shelf Program. CA-DL1 and CA-TVC were supported by the Natural Science and Engineering Council of Canada Discovery Grant program through grants awarded to P.M.L., E.R.H. and O.S. and the Natural Science and Engineering Council of Canada Discovery Grant program through grants awarded to P.M.L., E.R.H., and O.S.
